# Anaesthesia for Awake Fiberoptic Intubation: Ultrasound-Guided Airway Nerve Block versus Ultrasonic Nebulisation with Lignocaine

**DOI:** 10.15190/d.2021.4

**Published:** 2021-03-31

**Authors:** Jharana Mohanta, Ajit Kumar, Ashutosh Kaushal, Praveen Talawar, Priyanka Gupta, Gaurav Jain

**Affiliations:** ^1^Department of Anaesthesiology, All India Institute of Medical Sciences, Rishikesh, Uttarakhand, India; ^2^Department of Anaesthesiology, All India Institute of Medical Sciences, Bhopal, M.P., India

**Keywords:** Difficult airway management, awake fiberoptic intubation, ultrasound, airway nerve blocks, lignocaine, ultrasonic nebulizer.

## Abstract

Background: In anticipated difficult airway, awake fiberoptic guided intubation should be the ideal plan of management. It requires sufficient upper airway anesthesia for patient’s comfort and cooperation. We compared the efficacy of ultrasound guided airway nerve blocks and ultrasonic nebulisation with lignocaine for airway anesthesia before performing awake fibreoptic guided intubation. Methods: This prospective, randomised study included sixty consenting adult patients of both genders (American Society of Anesthesiologists' physical status 1–3) with anticipated difficult airway undergoing surgery. Ultrasound guided airway nerve blocks group received ultrasound-guided bilateral superior laryngeal (1 ml of 2% lignocaine) and transtracheal recurrent laryngeal (2 ml of 2% lignocaine) airway nerve blocks and ultrasonic nebulisation with lignocaine group received ultrasonic nebulisation of 4 ml of lignocaine 4%. The primary outcome was to compare the time required to intubate, whereas the secondary outcomes were to compare cough reflex and gag reflex, hemodynamic changes, number of attempts required, and  comfort score during awake fibreoptic guided intubation in both the groups. Results: The time taken for intubation was significantly lower in the ultrasound guided airway nerve blocks group 69.27±21.85 s than ultrasonic nebulisation with lignocaine group 92.43 ± 42.90 s (p = 0.015). Hemodynamic variables changed during the procedure but the values were comparable in both groups. There were no statistical differences in cough and gag reflexes, number of attempts, and comfort score in both groups. Conclusions: This study shows that significant lesser time required for performing awake fiberoptic intubation when patient received ultrasound guided airway nerve block in comparison to ultrasonic nebulisation for airway anaesthesia.

## INTRODUCTION

Awake fiberoptic guided intubation is now considered as the gold standard modality for difficult airway management^1,2^. Since awake fiberoptic nasotracheal intubation causes discomfort to the patients, it is essential to anaesthetize the upper airway sufficiently before performing awake fibreoptic guided intubation to ensure patient’s comfort and cooperation by preventing cough and gag reflexes and undesirable hemodynamic changes^3^. Due to anatomical variations, ultrasound guided airway blocks are the better option for anaesthetizing the upper airway. It requires small doses of local anaesthetic so there will be lesser chances of systemic toxicity. Ultrasound guided blocks are supposed to reduce unnecessary blind pricks, prevents vessel puncture, and hematoma formation^4^. Ultrasonic nebulisation of local anaesthetics is another technique that deposits ultrasonic mists of a local anaesthetic directly over the mucosa, thus anaesthetizing nasopharynx, oropharynx, laryngopharynx. Furthermore, this technique does not require detailed knowledge of anatomy, specialist skills, or experience. It can also be used in cases of massive neck swelling where nerve blocks are difficult to performed. The requirement for large doses of local anaesthetic (due to wastage during administration), and a higher chance of failure are some of the other disadvantages that need to be weighed against the benefits associated with it^5^. Literature has mainly focused on the comparison of blind block with lignocaine versus nebulisation with lignocaine^2^. However, recently ultrasound guided nerve block has gained popularity^6^. Thus, we planned to compare ultrasonic nebulisation with lignocaine and ultrasound guided airway nerve blocks to identify better methods of anaesthetizing upper airway for awake fiberoptic intubation. The primary objective was to compare the time required to intubate, whereas the secondary objectives were to compare cough reflex and gag reflex, hemodynamic changes, number of attempts required and comfort score during the awake fiberoptic guided intubation procedure in both the groups.

## MATERIALS AND METHODS

After approval from Institutional Ethics Committee Science, this prospective, randomized study was conducted. The study protocol is registered prospectively with the Clinical Trial Registry - India (CTRI/2019/08/020676). Based on the data from previous studies^2^, and the objectives of this study, the sample size calculated was 50, according to the following method: n = (Zα/2+Zβ)2 * (p1(1-p1)+p2(1-p2)) /(p1-p2)2, where Zα/2 is the critical value of the Normal distribution at α/2 (e.g. for a confidence level of 95%, α is 0.05 and the critical value is 1.96), Zβ is the critical value of the Normal distribution at β (e.g. for a power of 80%, β is 0.2 and the critical value is 0.84) and p1 and p2 are the expected sample proportions of the two groups 64% Vs 24%. With ~20% dropout in each arm total sample taken was 60.

**Figure 1:**
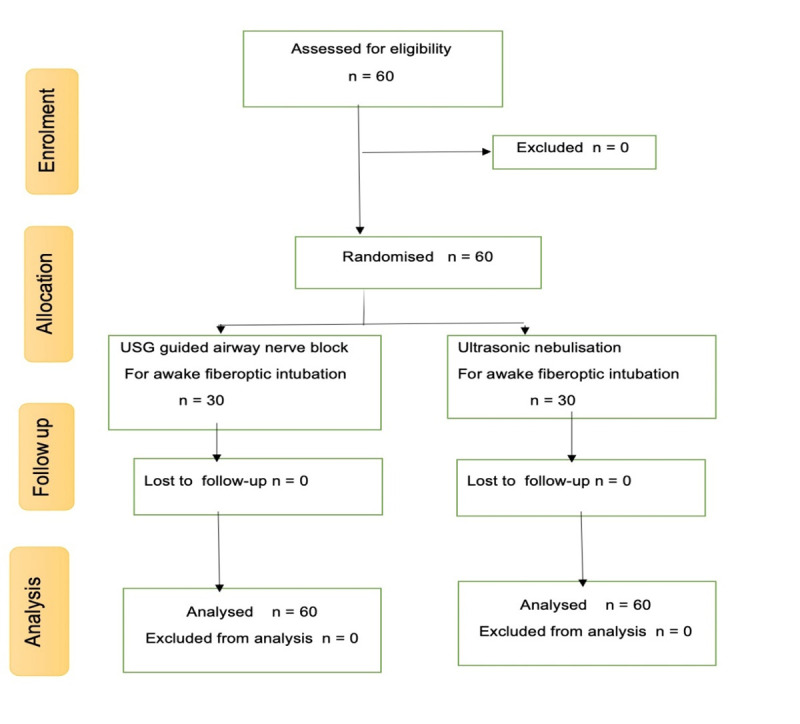
Consort Diagram

The study was conducted between 12/08/2019 to 15/06/2020. After obtaining written informed consent, 60 patients between 18–65 years of both genders, with American Society of Anaesthesiologists (ASA) physical status of I–III, with anticipated difficult airway (Modified Mallampati Score 3,4) and undergoing surgery under general anaesthesia were included. Patients who did not give consent, had an allergy to any study drug, asthmatic, epileptic, hemodynamically unstable, had blood in the oral cavity, glottic or subglottic obstructions, or had a deranged coagulation profile were planned to be excluded from the study. However, no patient met exclusion criteria. Figure 1 denotes the consort diagram.

A complete preoperative evaluation was performed. In the airway evaluation, mouth opening Modified Mallampati grading, thyromental distance, temporomandibular joint mobility, and dentition were assessed. History regarding the illness, past surgeries, and comorbidities were noted. The awake fiberoptic guided intubation procedure was explained to the patient. Standard fasting protocol and anti-aspiration prophylaxis with tablet ranitidine 150 mg orally were prescribed.

The patients were randomly allocated into two groups of 30 each by randomization, using computer generated randomization number. The ultrasound guided nerve blocks (UAB) group (n = 30) received ultrasound-guided airway nerve block and the ultrasonic nebulisation with lignocaine (LA) group (n = 30) received ultrasonic nebulization.

Intravenous (IV) line was secured preoperatively. Baseline hemodynamic parameters, such as the mean arterial pressure, heart rate, and pulse oximetry were recorded in the preoperative room. After shifting the patient to the operating room, monitors (ECG, pulse oximeter, blood pressure) were attached to the patient at the supine position and baseline value were noted. Injection with glycopyrrolate 0.2 mg IV was given to decrease airway secretions and 2 drops of xylometazoline decongestant nasal drop was put in bilateral nostrils of all patients 10 minutes before the procedure. Injection midazolam 1mg IV was given 5 minutes before the procedure to all the patients. Patients were divided into two groups by randomization using computer generated randomization number. Group UAB (n = 30) received ultrasound (Vivid iq GE medical systems, China) guided bilateral superior laryngeal nerve block and transtracheal block.

**Figure 2:**
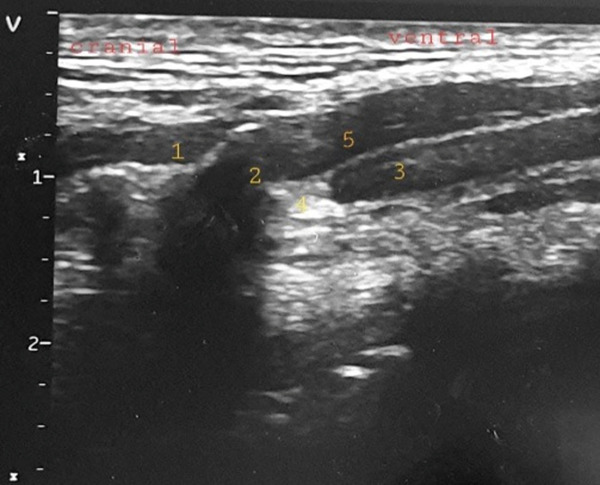
Sonography of left sub-hyoid region 1 – hyoid bone, 2 – thyrohyoid membrane, 3 – thyroid cartilage, 4 – internal branch of the superior laryngeal nerve, 5 – thyrohyoid muscle)

**Figure 3:**
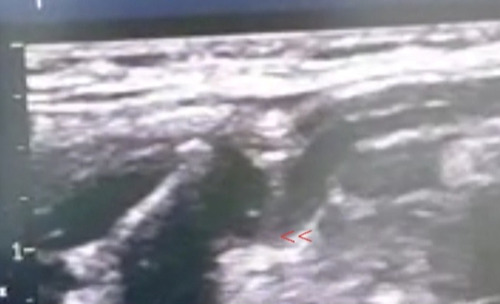
Needle at internal branch of the superior laryngeal nerve

In ultrasound guided airway nerve block the linear probe of frequency 6–13 MHz was placed over the submandibular area with parasagittal orientation and an out of plane approach was employed. The greater cornua of hyoid bone and thyroid cartilage were identified as hyperechoic structures on sonography. The thyrohyoid muscle and thyrohyoid membrane were appreciated between these two structures. The cricothyroid membrane lies between the caudal border of the thyroid cartilage and the cephalad border of the cricoid cartilage. The internal branch of the superior laryngeal nerve which commonly pierces the thyrohyoid membrane was blocked by administrating 1 ml of 2% lignocaine between greater horn of hyoid and thyroid cartilage just above thyrohyoid membrane on each side with 23G hypodermic needle (Figure 2 and Figure 3). Transtracheal injection with 2 ml of 2% lignocaine between the thyroid cartilage and cricoid ring was administered through the cricothyroid membrane placing the probe in the midline with transverse scan. Aspiration of air confirms trachea.

Group LA (n = 30) received 4 ml of 4% lignocaine by an ultrasonic nebulizer (Atom Sanitizer 303 – Atom Medical Corp. Japan)^7^for 10 min before the procedure.

Adequate effect of local anaesthesia was confirmed by the heaviness of tongue in Group LA patients and by hoarseness of voice in Group UAB patients. Awake fibreoptic guided intubation was performed while giving supplemental oxygen through nasal prongs. Appropriate sizes of endotracheal tube were used for both groups of patients (8.0 mm internal diameter for male and 7.0 mm internal diameter for female).

Time taken for intubation from the insertion of fiberoptic to the final railroading of the endotracheal tube during insertion of fiberoptic bronchoscope were the primary outcome whereas cough reflexes and gag reflexes score (Table 1)^8^, the hemodynamic changes (at baseline, during intubation, immediately after intubation, at 5 min after intubation, at 10 min after intubation), number of attempts required, position of vocal cords, and comfort score were secondary outcomes. For patients having cough and gag score ≥2 or more in our study were received 2 puffs of lignocaine 10% spray for gag reflex and 1 ml of 4% lignocaine through instrument channel port of fiberscope for cough reflex in both the groups.

**Table 1:** Cough and gag score, comfort score

Factors	Score
Cough and gag score	1. None
	2. Minimal coughing and gagging <3 times
	3. Mild cough and gag lasting for >3 times, <1 min
	4. Persistent coughing and gagging
	5. Need of rescue topical anesthesia
Comfort score	1. Excellent, calm patient
	2. Good, comfortable patient
	3. Moderately comfortable, need to pacify the patient
	4. Poor, uncomfortable
	5. Agitated

After the airway was secured, general anaesthesia was administered with injection fentanyl 2 mg/kg IV, injection propofol 2 mg/kg IV, and injection vecuronium 0.1 mg/kg IV. Patient comfort score was assessed during awake fiberoptic guided intubation. Signs of lignocaine toxicity (ECG changes, seizures, and bronchoconstriction) if any were noted.

The data analysis was done using Statistical Package for Social Sciences (SPSS) version 21.0. Categorical variables were presented in number and percentage (%) and continuous variables were presented as mean ± SD. The normality of data was tested by the Kolmogorov-Smirnov test. If the normality was rejected, then a non-parametric test was used. Quantitative variables were compared using the Independent t-test/Wilcoxon test (when the data sets were not normally distributed) between the two groups. Post hoc pairwise comparison for the Friedman test was done using the Nemenyi test for comparison within-group across the time period. Qualitative variables were correlated using the Chi-Square test/Fisher’s Exact test. A p-value of <0.05 was considered statistically significant.

## RESULTS

Sixty patients who meet the inclusion criteria were enrolled and analysed. Demographic data were similar between the two groups. There was no significant difference between the groups in terms of age, sex, body weight, body mass index (BMI) or American Society of Anaesthesiologist physical status. The groups were also comparable in terms of airway difficulty (Table 2, Table 3).

**Table 2:** Association between Group and Demographic Parameter Body Mass Index - BMI; TMD - thyromental distance.

Parameters Assessed	Group: UAB (n = 30)	Group: LA (n = 30)	p-value
GENDER			0.176
Males	22 (73.3%)	17 (56.7%)	
Females	8 (26.7%)	13 (43.3%)	
Height (cm)	157.47 ± 5.75	154.57 ± 5.92	0.059
Weight (kg)	60.50 ± 11.04	58.23 ± 8.81	0.383
BODY MASS INDEX (BMI)			0.670
BMI <18.5 kg/m2	3 (10.0%)	1 (3.3%)	
BMI = 18.5-24.9 kg/m2	15 (50.0%)	18 (60.0%)	
BMI = 25.0-29.9 kg/m2	10 (33.3%)	8 (26.7%)	
BMI >30 kg/m2	2 (6.7%)	3 (10.0%)	
Age (Years)	37.40 ± 14.60	43.20 ± 14.31	0.118
ASA GRADING			0.398
ASA Grade: I	17 (56.7%)	17 (56.7%)	
ASA Grade: II	9 (30.0%)	12 (40.0%)	
ASA Grade: III	4 (13.3%)	1 (3.3%)	
Mouth Opening (cm)	1.90 ± 0.80	2.17 ± 0.87	0.285
Modified Mallampatti Score	3.40 ± 0.56	3.10 ± 0.76	0.128
TMD (thyromental distance)			0.706
TMD < 6 cm	3 (10.0%)	5 (16.7%)	
TMD > 6 cm	27 (90.0%)	25 (83.3%)	
DENTITION			0.519
Normal dentition	25 (83.3%)	23 (76.7%)	
Protruded dentition	5 (16.7%)	7 (23.3%)	

**Table 3:** Quality of Airway Anesthesia based on four Parameters Assessment

Parameters Assessed	Group UAB (n=30)	Group: LA (n=30)	p-value
1. Attempts Required for awake fiberoptic guided intubation			0.233
1 attempt	24 (80.0%)	20 (66.7%)	
2 attempts	6 (20.0%)	10 (33.3%)	
			
2. Cough Gag Score	1.33 ± 0.55	1.57 ± 0.77	0.284
Score - 1	21 (70.0%)	18 (60.0%)	
Score - 2	8 (26.6%)	7 (23.3%)	
Score - 3	1 (3.3%)	5 (16.6%)	
			
3. Time taken for intubation (seconds)	69.27 ± 21.85	92.43 ± 42.90	0.015
			
4. Comfort Score	1.30 ± 0.60	1.37 ± 0.56	0.475
Score - 1	23 (76.6%)	20 (66.6%)	
Score - 2	5 (16.6%)	9 (30.0%)	
Score - 3	2 (6.6%)	1 (3.3%)	

Awake fiberoptic guided intubation was completed in all patients in both groups. The time taken to perform awake fibreoptic intubation was less in Group UAB (69.27±21.85s) than Group LA (92±42.90s) and was statistically significant (p= 0.015). The average value of cough and gag score in Group UAB and Group LA were 1.33±0.55 and 1.57±0.77 respectively (p-value 0.284).

**Figure 4:**
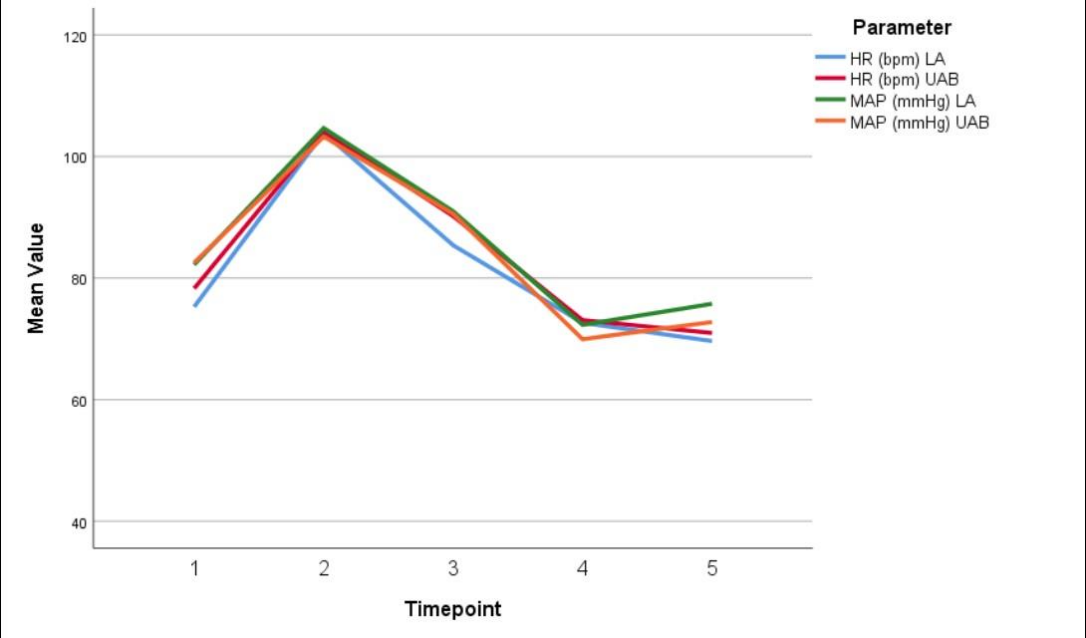
Hemodynamic changes at different time points 1 - preop, 2 - during intubation, 3 - immediate after intubation, 4 - at 5min after intubation, 5 – at 10 min after intubation.

Haemodynamic variables (heart rate, mean arterial pressure)were statistically comparable in both groups at all 5 points preoperatively, during awake fibreoptic guided intubation, immediate post-intubation, 5 min post-intubation and 10 min post-intubation (Figure 4). Signs or symptoms of lignocaine toxicity were not found at any point of time during or after the procedure.

## DISCUSSION

The mean time taken for intubation was significantly less in the ultrasound guided nerve block group as compared to the ultrasonic nebulisation group in the present study, which is similar to the findings of various studies^2,8,9^. The better airway block in ultrasound guided nerve block group might be the reason of lesser time requirement in this group in comparison to other group. However, few other studies^5,9^reported similar time taken to intubate.

In our study, mean cough and gag score in the UAB group was statistically similar to that of the LA group, though the number of cough and gag episodes are higher in Group LA. Studies^2,8,9^having similar finding in terms of higher cough and gag scores and increased coughing and gagging episodes in nebulization group than nerve block group. Suboptimal raining down effect of local anaesthetic into the trachea during atomization might have been the reason for lesser comfort observed in the patients of the atomization group^9^_._

There was no significant difference in the trend of heart rate and mean arterial pressure in both groups in the present study. Gupta et al^2^, also found that there was no statistically significant difference between both groups at any interval for heart rate or blood pressure. Vasu et al^8^_,_ also reported that at baseline values of heart rate and mean arterial pressure were also comparable in their study when compared atomized lignocaine nebulisation and transtracheal injection of lignocaine. However, in their study, after sedation, the heart rate was noted to be significantly high during the transtracheal injection. Heart rate was comparable in both groups. In contract, Singh et al^10^found that intra-group comparison of heart rate, with readings, obtained 1 and 3 minutes after awake fiberoptic intubation, revealed a significant increase in heart rate in patients received nebulization, which returned to near baseline in subsequent readings taken after intubation. However, there were no significant intergroup differences in the changes in heart rate from baseline at any point during intubation. The hemodynamic stability in both groups in present study indicates that airway was adequately blocked in both the groups.

Lesser attempt and better patient comfort score in UAB group is also indicating better quality of airway anesthesia. Few other studies also supported this result although they compare blind airway block with nebulization^5,8,9^. One recent similar study concluded that airway anesthesia using airway nerve blocks is superior to lignocaine nebulization for awake fiberoptic bronchoscopy-guided nasotracheal intubation, in terms of ease of intubation, less intubation time and patient comfort and satisfaction. But in that study authors used jet nebulization and blind airway nerve block whereas in present study ultrasonic nebulisation and ultrasound guided nerve block was used^11^.

All patients having a gag and cough reflex score ≥ 2 (9 patients in Group UAB and 12 patients in Group LA) received supplemental 2 puffs of lignocaine 10% spray for gag reflex and 1 ml of 4% lignocaine instillation for cough reflex in both the groups. Maximum amount of lignocaine given in UAB Group was 128 mg and 213.5 mg in LA Group, within the safe limits (5 mg/kg); no signs and symptoms of lignocaine toxicity were seen. To avoid the risk of respiratory depression and subsequent hypoxia in patients with anatomically compromised airway, sedative agents were not administered.

Few other studies make comparison between blind airway block to nebulisation for awake fiberoptic guided intubation, whereas in this study we aimed to compare the ultrasound guided airway block with ultrasonic nebulisation and this is the uniqueness as well as the strength of the present study.

Our study has several limitations too, being an unblinded study and having the serum lignocaine levels not measured.

## CONCLUSION

In comparison to ultrasound guided nerve blocks, ultrasonic lignocaine nebulisation took significantly more time for intubation, without any significant differences in the cough gag reflex, ease of intubation, hemodynamic changes, and postoperative complications. This study is important for the international medical scientific community recommending the ultrasound guided airway block whenever feasible for awake fibreoptic guided intubation.

## KEY POINTS

**◊** *Significant less time required to perform awake fibreoptic intubation in patients undergoing ultra-sound guided airway nerve block in comparison to ultrasonic nebulisation for airway anaesthesia.*

**◊** *There are no significant differences in the cough gag reflex, ease of intubation, hemodynamic changes, and postoperative complications in patients undergoing awake fiberoptic intubation when patient received ultrasound guided airway nerve block in comparison to ultrasonic nebulisation for airway anaesthesia.*

**◊** *Ultrasound guided airway block should be preferred over ultrasonic nebulisation to achieve airway anaesthesia.*
